# A case of incisional hernia repair using Composix mesh prosthesis after antethoracic pedicled jejunal flap reconstruction following an esophagectomy

**DOI:** 10.1186/s40792-017-0353-8

**Published:** 2017-06-29

**Authors:** Atsushi Yasuda, Takushi Yasuda, Hiroaki Kato, Mitsuru Iwama, Osamu Shiraishi, Yoko Hiraki, Yumiko Tanaka, Masayuki Shinkai, Motohiro Imano, Yutaka Kimura, Haruhiko Imamoto

**Affiliations:** 1 Department of Surgery, Kindai University Faculty of Medicine, 377-2 Ohno-higashi, Osaka-Sayama, Osaka 589-8511 Japan; 2 Cancer Center, Kindai University Hospital, 377-2 Ohno-higashi, Osaka-Sayama, Osaka 589-8511 Japan

**Keywords:** Incisional hernia, Mesh repair, Esophagectomy, Antethoracic pedicled jejunal flap reconstruction

## Abstract

**Background:**

An incisional hernia in a case of antethoracic pedicled jejunal flap esophageal reconstruction after esophagectomy is a very rare occurrence, and this hernia was distinctive in that the reconstructed jejunum had passed through the hernial orifice; a standard surgical treatment for such a presentation has not been established. Herein, we describe a case of repair using mesh prosthesis for an atypical and distinctive incisional hernia after antethoracic pedicled jejunal flap esophageal reconstruction.

**Case presentation:**

A 77-year-old woman with a history of subtotal esophagectomy who had undergone antethoracic pedicled jejunal flap reconstruction complained of epigastric prominence and discomfort without pain. On examination, she had an abdominal protrusion between the xiphoid process and the umbilicus that contained the small bowel. Computed tomography showed that the fenestration of the abdominal wall that was intentionally created for jejunum pull-up was dehisced in a region measuring 9 × 15 cm and the small intestine protruded through it into the subcutaneous space without strangulation. Because the hernial orifice was too large and the reconstructed jejunum was passing through the hernial orifice in this case, we applied a parastomal hernia repair method that was modified from the inguinal hernia repair using the Lichtenstein technique. After 3 years and 5 months following surgery, the patient has recovered without hernia recurrence or other complications.

**Conclusion:**

We consider this to be the first case of repair using Composix mesh prosthesis for repair of an atypical and distinctive incisional hernia after an antethoracic pedicled jejunal flap reconstruction. This method seems to be useful and could potentially be widely adopted as the surgical treatment for this condition.

## Background

The overall reported occurrence of incisional hernias post laparotomy varies within a range between 11 and 23% in different studies [1–3]. However, an incisional hernia in a case of antethoracic pedicled jejunal flap esophageal reconstruction is a very rare occurrence. Antethoracic jejunal esophageal reconstruction—an alimentary tract reconstructive method following esophagectomy—is conducted only in patients who have undergone gastric surgery previously. The distinctive feature of an incisional hernia in such patients is that the pulled-up jejunum passes through the hernial orifice together with the herniating intestine. Incisional hernias can be conventionally repaired by two methods: simple re-suturing or repair with mesh prosthesis; however, it is recommended that incisional hernias measuring more than 10 cm in diameter be repaired with mesh prosthesis because of the high recurrence rate with simple re-suturing [4, 5]. However, a mesh prosthesis cannot be conventionally used in an incisional hernia after antethoracic jejunal reconstruction, and consequently, the standard surgical treatment for this condition remains unclear.

We, herein, describe our clinical experience of a case of incisional hernia, which developed after a postesophagectomy antethoracic pedicled jejunal reconstruction and was successfully repaired with our originally modified parastomal hernial repair method using a mesh prosthesis.

## Case presentation

A 77-year-old woman who had undergone distal gastrectomy (Billroth I) for early gastric cancer 3 years earlier then underwent a subtotal esophagectomy for esophageal squamous cell carcinoma that was followed by esophageal reconstruction with an antethoracic pedicled jejunal flap using the supercharge technique. No postoperative complication, except grade 1 surgical site infection according to the Clavien–Dindo classification [6, 7], occurred throughout the postoperative course. For the last 8 years, the patient had chronic constipation caused by a lumbar intervertebral disk herniation, and usually strained to pass stool, with resultant high abdominal pressure.

**Fig. 1:**
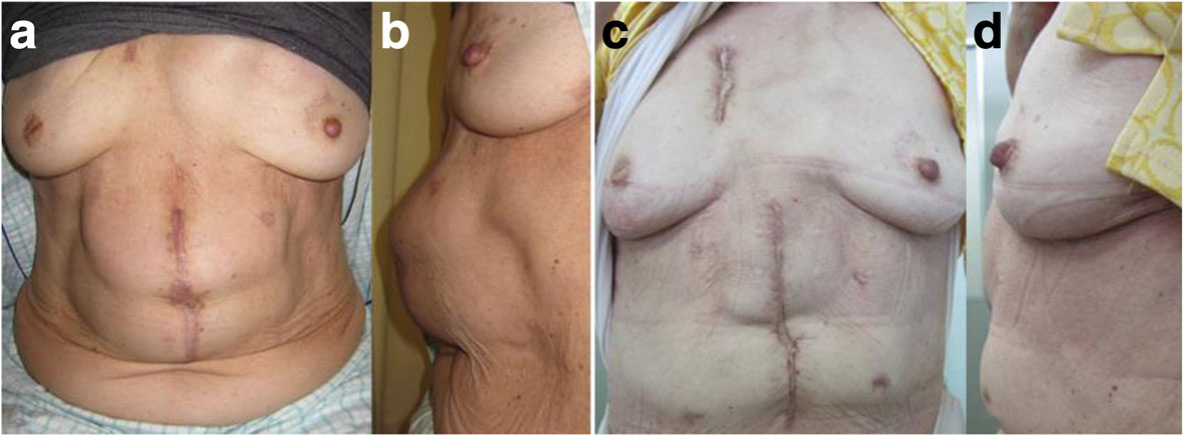
Physical examination before operation (**a**, **b**) and after operation (**c**, **d**)

**Fig. 2:**
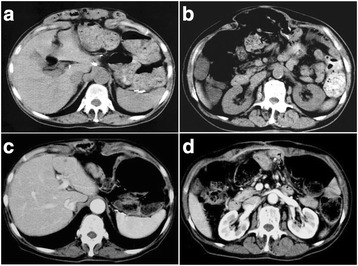
CT scan before and after surgery. **a**, **b** A large hernia with prolapsed small intestine was observed before surgery. **c**, **d** Mesh sheet covering the hernial orifice

Four months after the surgery, the patient complained of distention and discomfort without tenderness in the epigastric region. On examination, her vital signs were normal, but she had abdominal distention extending from the xiphoid process to the umbilicus (Fig. 1a, b). No other abnormalities were present on examination, and results of blood tests were normal. Computed tomography showed a herniation of the small intestine, measuring 9 × 15 cm, surrounding the pedicled jejunum, but without strangulation (Fig. 2a, b). As the size of the hernial orifice was increasing, conservative therapies, such as a hernia band, seemed to be contraindicated because of possible compression of the pedicled jejunum. Thus, surgical treatment was considered necessary.

### Surgical technique

Because the hernial orifice was large and the abdominal wall around it was thin and fragile, re-suturing would potentially provide insufficient strength for the abdominal wall to resist abdominal pressure. Therefore, we decided to reinforce the abdominal wall with the mesh repair method. A 14 × 18 cm large composite mesh that composed of two layers—polypropylene mesh and expanded polytetrafluoroethylene (Gore-Tex), named Composix Kugel Patch (C.R. Bard Inc.)—was selected as the mesh prosthesis. We applied a parastomal hernia repair technique—the keyhole technique [8–10], which is a modification of the Lichtenstein technique for inguinal hernia repair.

**Fig. 3:**
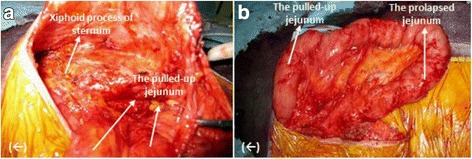
*(←)* cranial. **a**, **b** The distal portion of the pulled-up jejunum including the prolapsed jejunum was carefully dissected and exposed

In the supine position, the patient was administered general anesthesia, a median incision was made over the hernia, and scar revision was completed. Then, the edge of the incision was retracted upward, and the subcutaneous fat was carefully dissected to create a skin flap over and around the distal portion of the pulled-up jejunum (Fig. 3a). Then, the distal portion of the pulled-up jejunum was completely exposed (Fig. 3b), and the prolapsed jejunum was carefully separated from the area for complete exposure of the hernial orifice.

**Fig. 4:**
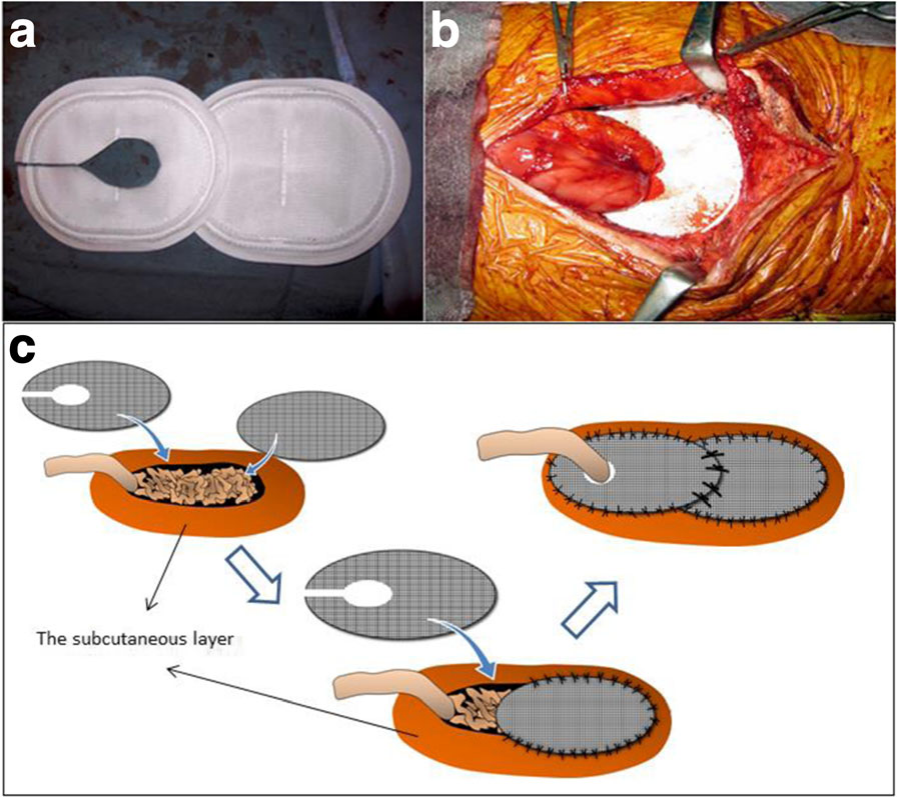
**a**–**c** We prepared two pieces of the mesh and applied a parastomal hernia repair technique that was a modified inguinal hernia repair procedure using the Lichtenstein technique—the keyhole technique

Because a piece of 14 × 18 cm Composix Kugel Patch was too small to cover the attenuated area around the orifice, we prepared two pieces of the mesh (Fig. 4a, c). The mesh was stretched out to the original shape by the elasticity of a shape-memory ring, which was stitched into the mesh immediately after the inferior edge of it was rolled up and introduced to the subcutaneous layer. The mesh was positioned on the inferior two thirds of the hernial orifice overlapping the fragile abdominal wall around the orifice. Interrupting sutures of 3–0 Vicryl at intervals of 1.0 cm along the edge of both the mesh and the orifice secured the mesh to the abdominal wall.

**Fig. 5:**
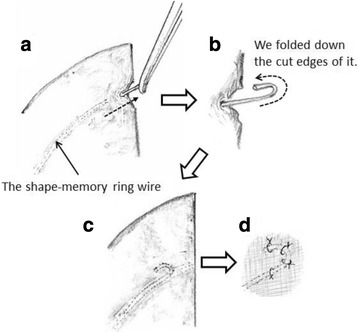
The processing schema of the cut edges of the shape-memory ring wire. **a**, **b** We pulled the shape-memory ring wire out from the cutting line of the mesh and folded down the cut edges of the shape-memory ring wire. **c**, **d** Then, we pulled back the wire in the mesh and fixed it tightly by several sutures

The other mesh was cut with a lateral slit and a medial blunt oval shape for the pulled-up jejunum. The manufacturer does not recommend making incisions on the Composix Kugel Patch (C.R. Bard Inc.), because reshaped patches may cause complications such as bowel perforation or surgical site infection; therefore, the cut edges of the shape-memory ring wire were folded down and fixed tightly by several sutures to prevent tissue injuries (Fig. 5a–d). After the mesh was positioned on the superior one third of the hernial orifice encircling the pulled-up jejunum, it was sutured to the abdominal wall and the orifice similarly as the inferior mesh (Fig. 4b, c). Then, we anchored the superior mesh to the inferior one by placing several sutures on the overlapping edges of the two meshes.

To prevent jejunal prolapse and seroma formation in the subcutaneous space, we approximated the skin flap and the surface aspect of the polypropylene mesh with 3–0 Vicryl sutures at intervals of 1.0 cm. Moreover, we placed tight sutures between the skin flap and the expanded polytetrafluoroethylene (ePTFE) side of the mesh at the edge of the oval margin circumferentially to keep the serous membrane of the reconstructed jejunum away from the surface of the polypropylene mesh. This procedure was undertaken to prevent adhesion between the mesh and the reconstructed jejunum. Two catheters were introduced into the subcutaneous space and attached to a closed system of suction, followed by the usual approximation of the subcutaneous tissue and skin closure.

### Postoperative course

The patient had an uneventful recovery postoperatively and was discharged without complications. Although she continues to suffer from constipation, her oral intake has been good and without discomfort. No hernia recurrence or complications have occurred in the 3 years and 5 months since the surgical intervention (Figs. 1c, d and 2c, d).

## Discussion and conclusions

To our knowledge, this is the first case to use a mesh prosthesis for repair of an incisional hernia that developed after a subtotal esophagectomy with antethoracic reconstruction by a pedicled jejunal flap. According to a national cancer registry in Japan, reconstructions using the jejunum accounted for 5.4% of all esophageal cancer resections [11]. The incidence of incisional hernia after this surgery was unclear.

In this case, we consider the following two factors to be responsible for wound dehiscence: one was fragility of the abdominal wall that resulted from a surgical site infection following a previous operation, and the other was the high abdominal pressure when straining to pass stool. A simple primary closure, therefore, seemed insufficient for surgical treatment. Moreover, the hernial orifice in this case was huge, and the abdominal wall around the orifice was weak; therefore, we decided to repair the hernia using a mesh prosthesis. In such a case of incisional hernia, with the pulled-up jejunum passing through the hernial orifice, a mesh prosthesis is required not only to reinforce the abdominal wall but also to allow the pulled-up jejunum to pass through the mesh without disturbing food passage. Therefore, we decided on a parastomal hernia repair technique using a mesh sheet, particularly the keyhole repairing technique [8–10], for this case. This surgical technique using mesh, frequently reported to be effective since Rosin and Bonardi developed it [8], is superior to the suture repairing technique with regard to recurrence [rate (range) 17.2% (11.9–23.4%) vs 69.4% (59.7–78.3%)] and wound infection [rate (range) 1.9% (0.4–5.5%) vs 11.8% (6.1–20.2%)] [12].

There is an another famous parastomal hernia repair technique, the Sugarbaker method, in which no hole is made in the mesh but the bowel going to the stoma is lateralized and covered by the mesh. It is desirable to tightly adhere the subcutaneous tissues to the skin from the perspectives of infection, recurrence, and visualization, but in this method, the skin and mesh might be distorted and a gap might occur in the subcutaneous space; thus, we did not choose this method.

Bowel fistula is a postoperative complication that should be feared exceedingly, but Jänes et al. reported that it did not occur within 5 years of observation in their randomized trial [13]. Steele et al. reported a low incidence (3%) that was acceptable [14]. However, caution must be exercised with regard to this complication even if the rate is low because it is a consequence of placing the mesh in direct contact with the bowel and accounts for the hesitation with the use of this prosthesis.

Thus, in our case, we created a mesh prosthesis with a diameter that was a few millimeters larger than the diameter of the pulled-up jejunum and sutured it tightly between the skin flap and the mesh at the edge of the oval edge circumferentially to keep the jejunum away from the polypropylene mesh. Moreover, we thought that a composite mesh with a porous external surface to encourage tissue integration and a smooth microporous internal surface to prevent adhesions when placed in contact with viscera would be better than the usual polypropylene mesh for prevention of unintended adhesion and, therefore, used it in this case. However, there was a problem in that the manufacturer does not recommend incisions or cuts on the Composix Kugel Patch (C.R. Bard Inc.), because a reshaped patch might cause complications such as bowel perforation or surgical site infection. Therefore, in order to avoid these complications, we placed the reshaped mesh in the subcutaneous space instead of in the abdominal cavity to ensure the intestine was kept away from the mesh. Moreover, we folded down the cut ends of the shape-memory ring and fixed them to the mesh tightly with sutures to prevent tissue injury (Fig. 5).

Three years and 5 months have elapsed without hernia recurrence or complications since the operation. This parastomal hernia repair using a composite mesh seems to be a useful treatment for incisional hernias developing after esophagectomy with antethoracic pedicled jejunal flap reconstruction.
